# Relationship between thyroid tumor radiosensitivity and nuclear localization of DNA-dependent protein kinase catalytic subunit

**DOI:** 10.1093/jrr/rraa032

**Published:** 2020-06-22

**Authors:** Makoto Ihara, Kazuko Shichijo, Kiyoto Ashizawa, Katsuya Matsuda, Ryota Otsubo, Ichiro Horie, Masahiro Nakashima, Takashi Kudo

**Affiliations:** 1 Department of Radioisotope Medicine, Atomic Bomb Disease and Hibakusha Medicine Unit, Atomic Bomb Disease Institute, Nagasaki University, 1-12-4 Sakamoto, Nagasaki 852-8523, Japan; 2 Department of Tumor and Diagnostic Pathology, Atomic Bomb Disease and Hibakusha Medicine Unit, Atomic Bomb Disease Institute, Nagasaki University, 1-12-4 Sakamoto, Nagasaki 852-8523, Japan; 3 Department of Surgical Oncology, Nagasaki University Graduate School of Biomedical Sciences, 1-7-1 Sakamoto, Nagasaki 852-8588, Japan; 4 Department of Endocrinology and Metabolism, Nagasaki University Graduate School of Biomedical Sciences, 1-7-1 Sakamoto, Nagasaki 852-8588, Japan

**Keywords:** thyroid tumor, radiosensitivity prediction, DNA-PKcs, immunohistochemical staining

## Abstract

Thyroid tumors are the most common types of endocrine malignancies and are commonly treated with radioactive iodine (RAI) to destroy remaining cancer cells following surgical intervention. We previously reported that the expression levels of double-stranded DNA-dependent protein kinase catalytic subunit (DNA-PKcs), which plays a key role in non-homologous end joining, are correlated with the radiosensitivity of cancer cells. Specifically, cells expressing high levels of DNA-PKcs exhibited radiation resistance, whereas cells expressing low levels were sensitive to radiation treatment. In this study, we observed full-length native DNA-PKcs (460 kDa) in radiation-resistant FRO and KTC-2 cells through western blot analysis using an antibody against the C-terminus of DNA-PKcs. In contrast, cleaved DNA-PKcs (175 kDa) were observed in radiation-sensitive TPC-1 and KTC-1 cells. Almost equal amounts of DNA-PKcs were observed in moderately radiation-sensitive WRO cells. We also describe a simple method for the prediction of radiation therapy efficacy in individual cases of thyroid cancers based on staining for DNA-PKcs in human cancer cell lines. Immunofluorescent staining showed that native DNA-PKcs was localized largely in the cytoplasm and only rarely localized in the nuclei of radiation-resistant thyroid cancer cells, whereas in radiation-sensitive cancer cells a 175-kDa cleaved C-terminal fragment of DNA-PKcs was localized mainly inside the nuclei. Therefore, DNA-PKcs moved to the nucleus after γ-ray irradiation. Our results suggest a new method for classifying human thyroid tumors based on their cellular distribution patterns of DNA-PKcs in combination with their radiosensitivity.

## INTRODUCTION

Although thyroid tumor cells have been shown to incorporate radioactive iodine for clinical purposes, no studies have reported the relationship between the clinical endpoint and expression of double-stranded DNA-dependent protein kinase (DNA-PK) in thyroid tumor cells. Thyroid cancers are the most common endocrine malignancy and consist of three major types: papillary carcinoma, follicular carcinoma and anaplastic carcinoma. All of these malignancies are derived from thyroid follicular cells. Among them, papillary carcinoma are the most common (80–90%), followed by follicular carcinoma (5–10%) [1, 2]. However, the prognosis and treatment of thyroid cancers depend on the tissues involved. Although anaplastic carcinomas comprise only 1–3% of all thyroid cancers, they account for 14–50% of all thyroid cancer-related mortality [3].

Ionizing radiation induces multiple forms of DNA damage including highly cytotoxic double-stranded breaks (DSBs) [4, 5]. If left unrepaired or repaired incorrectly, DSBs induce mutations, chromosomal aberrations and cell death. In eukaryotes, DSBs are repaired primarily by homologous recombination (HR) or non-homologous end joining (NHEJ) [6, 7], the latter of which is the most common repair mechanism in mammalian cells. Moreover, DNA-PK plays an important role in the process of NHEJ. DNA-PK is a serine/tyrosine protein kinase composed of double-stranded DNA-dependent protein kinase catalytic subunit (DNA-PKcs) and the Ku70/80 heterodimer. Ku70/80 binds to DSB ends and recruits DNA-PKcs to form an active kinase complex [8]. The active DNA-PK complex then phosphorylates various repair proteins.

In our previous study, we reported that the expression of DNA-PKcs within thyroid cancer cells is correlated with the radiosensitivity of these cells [9]. In contrast, the expression of Ku70/80 is not correlated with radiosensitivity. These results suggest that cells expressing high levels of DNA-PKcs were resistant to radiation whereas those expressing low levels of DNA-PKcs were sensitive to radiation therapy.

If cancers are large and located within the thyroid, or if the cancer has spread to lymph nodes or other parts of the body, radioactive iodine (RAI) therapy is commonly used to destroy residual cancer cells following surgical interventions. However, no standardized method has been established for the administration of RAI therapy. RAI therapy after total thyroidectomy is generally performed using 30–100 mCi, and in cases of bone and/or lung metastasis, a dose of 100–200 mCi is administered in Japan [10]. Moreover, numerous adverse effects have been reported to be associated with RAI, including radiation damage to the salivary glands [11].

Identification of thyroid cells with low repair capacity is important for achieving highly efficient treatment. However, current predictive measures require extraction of sufficient amounts of protein from isolated tissue samples for quantification of intact DNA-PKcs by SDS-PAGE and western blotting analysis, which are time-consuming procedures.

Therefore, the aim of this study was to develop a simple and clear method to predict the effect of radiation in individual cases of thyroid tumors based on immunohistochemical staining of DNA-PKcs using tumor cells isolated for cytological analysis.

## MATERIALS AND METHODS

### Cell cultures

The human thyroid cells employed consisted of papillary carcinoma (TPC-1, KTC-1; radiation-sensitive), follicular carcinoma (WRO) and anaplastic carcinoma (FRO and KTC-2; radiation-resistant) cells [9]. Table 1 shows the characteristics of the cell lines. Cells were cultured in Dulbecco’s modified Eagle’s medium (high-glucose) and nutrient mixture F-12 HAM (Fujifilm Wako Pure Chemical, Doshomachi, Osaka, Japan) (1:1) supplemented with 5% fetal bovine serum (FBS; Equitech-Bio, Inc. Kerrville, TX, USA) in humidified 5% CO_2_ at 37°C.

**Table 1:** characteristics of the cell lines

Cell line	Cancer type	Radiation sensitivity
FRO	Anaplastic carcinoma	Resistant
KTC-2	Anaplastic carcinoma	Resistant
WRO	Follicular carcinoma	Moderate
KTC-1	Papillary carcinoma	Sensitive
TPC-1	Papillary carcinoma	Sensitive

### Antibody

A mouse monoclonal anti-DNA-PKcs antibody (Ab-2, clone: 25–4, Thermo Fisher Scientific, Waltham, MA, USA) recognizing the C- terminus of DNA-PKcs [12] was used in this study.

### Irradiation

Cells were irradiated with a ^137^Cs γ-irradiator (Pony Industry, Chuo-ku, Osaka, Japan) at a dose rate of 0.82 Gy/min at room temperature. To measure DSB repair, cells were irradiated with 20 Gy.

### Western blot analysis

Western blot analysis was performed as previously described [9]. Proteins were detected by incubating the blots with an anti-DNA-PKcs antibody (diluted 1:1000) at 4°C overnight.

### Immunofluorescent staining

Cells (1 × 10^5^) were seeded on 22 × 22-mm glass cover slips in 60-mm diameter culture dishes and incubated at 37°C for 2 days. After washing with PBS(−) (137 mM NaCl, 2.7 mM KCl, 10 mM Na_2_HPO_4_ and 1.76 mM KH_2_PO_4_, pH 7.4) three times, the cells were fixed with cold methanol (−20°C) for 10 min, and then incubated with anti-DNA-PKcs antibody (diluted 1:200). The primary antibodies were stained with Alexa Fluor 488 goat anti-mouse IgG (A-11001; Thermo Fisher Scientific) for 1 h at 37°C. Nuclei were stained with 4′,6-diamidino-2-phenylindole (DAPI) for 1 h at 37°C in the dark. Images were obtained via florescent microscopy (Olympus BX50, Shinjuku, Tokyo, Japan) at a magnification of ×400.

For immunofluorescent examination of cellular localization, the grades of DNA-PKcs were scored from ‘−’ to ‘+++’. DNA-PKcs staining was semi-quantitatively graded as the expression index according to a four-tiered scoring system: 0, negative (−); 1, weakly positive (+); 2, moderately positive (++); and 3, strongly positive (+++). A total of 100 cells per sample was counted and scored.

### Measurement of DNA DSB repair

The numbers of DSBs were calculated based on the density of bands observed after pulsed-field gel electrophoresis (PFGE) as previously described [13]. The gels were stained for 1.5 h with ethidium bromide (5 μg/mL) and destained for 3 h in 0.5× TBE buffer (44.5 mM Tris, 1 mM Ethylenediaminetetraacetic acid, 44.5 mM Boric acid). Fluorescence intensities were measured using a UV transilluminator from FluorChemR Imaging Systems (Alpha Innotech, San Leandro, CA, USA). The intensity of bands corresponding to fragmented DNA released from the origin was measured.

## RESULTS

### Western blot analysis

**Fig. 1.**
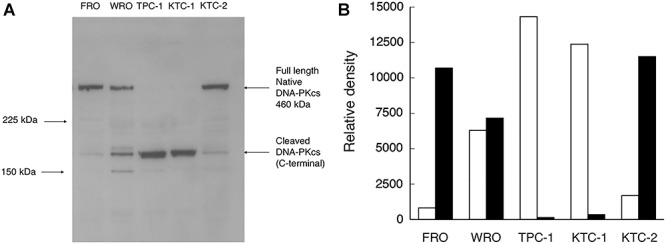
Expression of DNA-PKcs in thyroid cancer cells. (**A**) Western blotting results. Equivalent amounts of cell extracts (30 μg) from different thyroid cancer cell lines were analysed through western blotting. (**B**) Densitometric analysis of (A). Back bars, native DNA-PKcs (460 kDa); white bars, cleaved DNA-PKcs (170 kDa).

We quantified the level of DNA-PKcs by western blotting (Fig. 1A) and analysed the results by densitometry (Fig. 1B). Our results showed that although the same amount of protein (30 μg) was loaded into each well, the density of the full-length native DNA-PKcs (460 kDa) and cleaved DNA-PKcs (175 kDa) differed greatly. Moreover, the concentration of native DNA-PKcs band differed between the five different thyroid cancer cell lines. A clear native DNA-PKcs band was observed in radiation-resistant FRO and KTC-2 cells, whereas only a weak band was observed in radiation-sensitive TPC-1 and KTC-1 cells. Alternatively, high levels of the cleaved low-molecular weight fragment (175 kDa) were observed within radiation-sensitive TPC-1 and KTC-1 cells. No significant differences in the concentration of native or cleaved DNA-PKcs were observed in WRO cells, which exhibit moderate radiation sensitivity.

### Immunofluorescent staining of cultured thyroid cancer cell lines

**Fig. 2.**
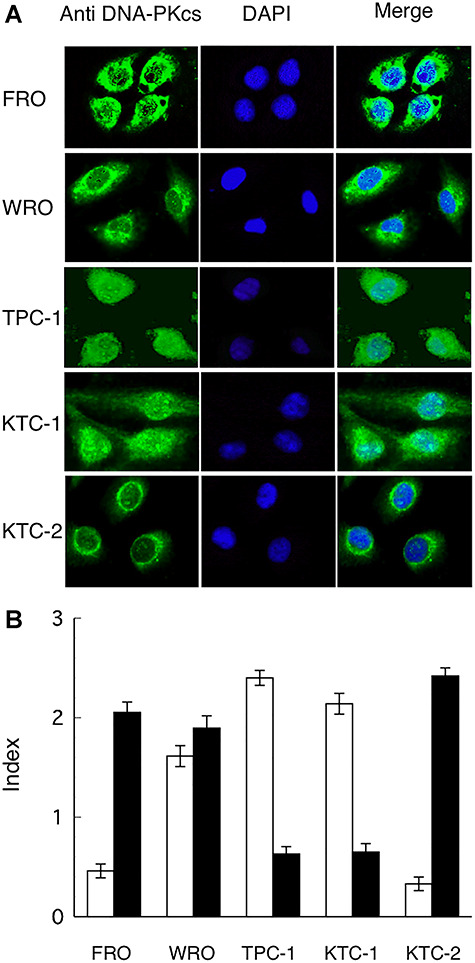
Localization of DNA-PKcs in thyroid cancer cells. (**A**) DNA-PKcs was detected by immunofluorescent staining in different thyroid cancer cell lines using primary antibodies specific for DNA-PKcs (Ab-2, clone 25–4). Original magnification ×400. (**B**) Semi-quantitative analysis of cellular localization of DNA-PKcs. DNA-PKcs expression detected with immunofluorescent staining was semi-quantitatively graded as an expression index according to a four-tiered scoring system: 0, negative (−); 1, weakly positive (+); 2, moderately positive (++); and 3, strongly positive (+++). White bars, in the nuclei; black bars, in the cytoplasm.

We then examined the cellular localization of the native and cleaved DNA-PKcs by immunofluorescent analysis. As shown in Fig. 2A, the cellular localization of DNA-PKcs differed between the radiation-resistant and radiation-sensitive cells. Within radiation-resistant cells (FRO, KTC-2), DNA-PKcs was observed in the cytoplasm and only rarely in the nuclei. In radiation-sensitive cells (TPC-1, KTC-1), DNA-PKcs was localized mainly in the nuclei but also in the cytoplasm. In the moderately radiosensitive WRO cells, DNA-PKcs was observed at a medium level both outside and inside of the nuclei (Fig. 2A).

Figure 2B shows the results of semi-quantitative analysis of cellular localization of DNA-PKcs. DNA-PKcs of radiation-sensitive thyroid cancer cells localized inside nuclei, whereas DNA-PKcs of radiation-resistant thyroid cancer cells localized in the cytoplasm.

### Relationship between expression index of DNA-PKcs located outside the nuclei and relative density of 460-kDa band

**Fig. 3.**
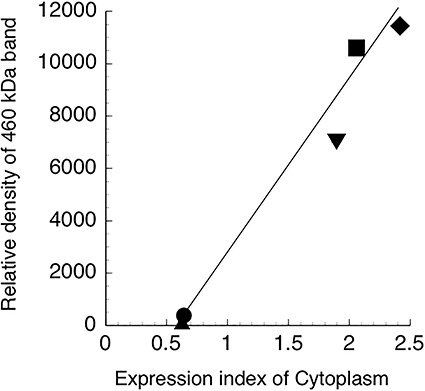
Relationship between expression index of DNA-PKcs localized in the cytoplasm and relative density of the native DNA-PKcs (460 kDa) signal. Diamond, KTC-2; square, FRO; inverted triangle, WRO; triangle, TPC-1; circle, KTC-1.

The relationship between the expression index of DNA-PKcs located outside the nuclei (Fig. 2B) and the relative density of the 460-kDa band density in western blotting (Fig. 1B) was examined. As shown in Fig. 3, a clear correlation was observed.

### Cellular distribution of DNA-PKcs after γ-ray irradiation

**Fig. 4.**
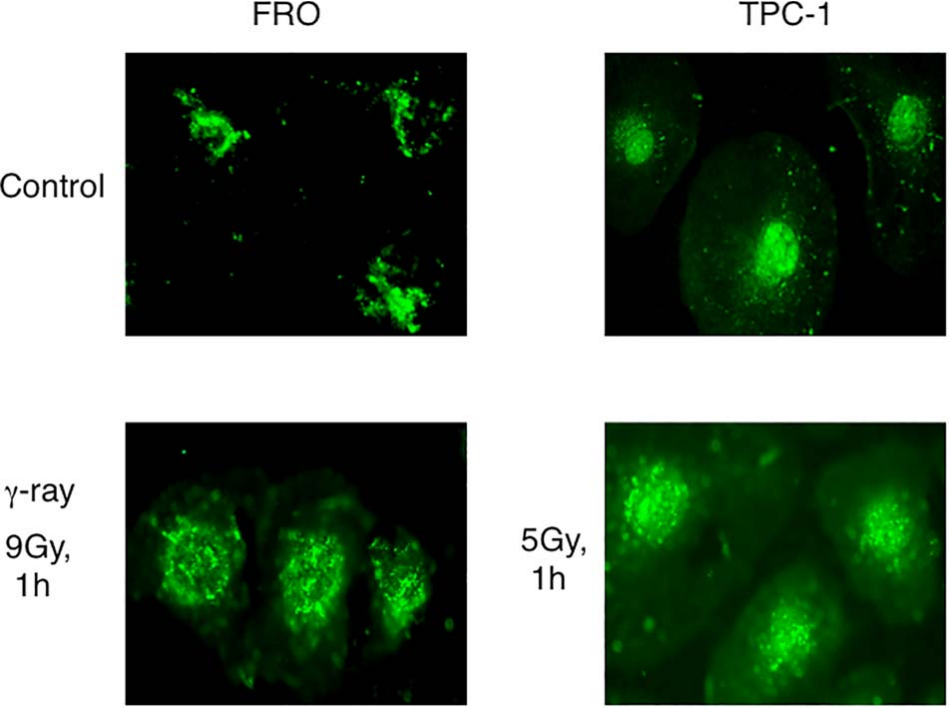
Localization of DNA-PKcs in FRO and TPC-1 cells without treatment and after 1 h of irradiation. FRO and TPC-1 cells were irradiated with 9 and 5 Gy, respectively.

As shown in Fig. 2, the cellular distribution of DNA-PKcs in non-irradiated cells was peculiar considering the function of this protein. To elucidate the function of DNA-PKcs, the cellular distribution of DNA-PKcs after γ-ray irradiation was examined. DNA-PKcs moved to the nucleus after γ-ray irradiation in FRO cells (Fig. 4). DNA-PKcs was also observed in the nuclei of TPC-1 cells. The number of foci increased after γ-ray irradiation in both radiation-resistant and radiation-sensitive cells.

### Repair of DSBs

**Fig. 5.**
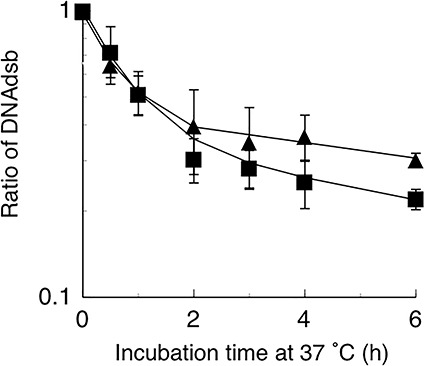
Repair of DSBs in FRO and TPC-1 cells using PFGE. Square, FRO; triangle, TPC-1.

The repair of DSBs after radiation in FRO and TPC-1 cells was examined by PFGE. As shown in Fig. 5, the repair of DSBs was biphasic. We estimated that 66 and 51% of DSBs were repaired by fast repair in FRO and TPC-1 cells, respectively.

## DISCUSSION

In our previous study, we found that FRO and KTC-2 cells are highly resistant to radiation, whereas TPC-1 and KTC-1 cells are sensitive to radiation and WRO cells exhibit moderate sensitivity. Further, we reported that the expression level of DNA-PKcs was correlated with radiation sensitivity [9]. As shown in Figs. 1 and 2, we found that the cleaved DNA-PKcs (175 kDa) was found primarily in radiation-sensitive cells (TPC-1 and KTC-1) and localized mainly inside of the cell nuclei. In contrast, full-length native DNA-PKcs (460 kDa) was expressed primarily in radiation-resistant cells (FRO and KTC-2) and was localized largely in the cytoplasm and only rarely in the nuclei. Moreover, nearly equal amounts of native and cleaved DNA-PKcs were observed in cells with moderate levels of radiation sensitivity (WRO). Many studies have shown that DNA-PKcs localizes in the nuclei under normal conditions [14, 15]. Koike *et al*. also reported that DNA-PKcs protein was found in the nuclear periphery of prophase cells [16]. However, Nagasawa *et al*. described that DNA-PKcs is present in the cytoplasm of quiescent peripheral blood mononuclear cells [17]. Gustafsson *et al*. reported that DNA-PKcs is localized outside the nuclei in mitotic cells [18]. DNA-PKcs was observed in the cytoplasm in non-treated bone marrow-derived macrophages [19]. Based on these results, the cellular distribution of the DNA-PKcs protein depends on the cell type, cycle and condition, DNA-PKcs activity and antibody used.

DNA-PKcs is a large protein (molecular weight 460 kDa). In apoptotic cells, DNA-PKcs is cleaved by a CPP32-like apoptotic protease [20] or ICE-like protease [21] to form 270- and 175-kDa fragments. Cleavage of the DNA-PKcs protein, which serves to separate the FAT/PIKK/FATC module from the DNA-binding HEAT repeats to result in loss of DNA-PK activity, was observed following treatment of cultured cells with pro-apoptotic agents. This mechanism may favor DNA fragmentation and nuclear disassembly necessary for apoptosis and confine DNA-PK activity to viable cells. Davis *et al*. reported that intact DNA-PKcs was cleaved into two large fragments, namely, an N-terminal fragment (N-PKcs; amino acids 1–2713; 300 kDa) and a C-terminal fragment (C-PKcs; amino acids 2714–4128; 175 kDa) [22]. We determined that the low-molecular weight band (175 kDa) observed in our study was the same as that observed for C-PKcs, and thus, within the thyroid cancer cells, the cleaved product of DNA-PKcs was determined to be C-PKcs. Davis *et al*. also found that N-PKcs, and not C-PKcs, was capable of localizing to DSBs in the nucleus *in vivo*, which further supports that N-PKcs is required for interaction with the Ku-DNA complex [22]. Furthermore, Uematsu *et al*. reported that DNA-PKcs accumulates at DSB sites in a Ku80-dependent manner [23]. However, the mechanism responsible for localization of the cleaved low-molecular weight C-PKcs to the nucleus requires further analysis.

Immunohistological analysis revealed a strong correlation between the radiosensitivity of human thyroid cancer cell lines and nuclear localization of DNA-PKcs (Fig. 2A and B). Further, these results agree with those obtained via western blot analysis (Fig. 1A and B). To confirm this, we examined the relationship between the expression index of DNA-PKcs located in the cytoplasm, outside of nuclei, and the expression level of native DNA-PKcs (460 kDa). As shown in Fig. 3, an almost linear regression curve was observed. These results are consistent with those of our previous report [9] and suggest a new method for the classification of human thyroid cancers based on the cellular distribution patterns of DNA-PKcs in combination with the radiosensitivity of the cells.

To confirm these hypotheses, the results of immunohistological staining of DNA-PKcs in human cytological specimens or tumor tissue samples must be correlated with radiation therapy outcomes in patients with diagnosed thyroid cancers; this provides a simple method for predicting the radiosensitivity of thyroid tumor cells. The radiation-sensitive cells (TPC-1 and KTC-1) used in this study are classified as papillary cancer cells. Ahmed *et al*. reported that the *D*_37_ values (radiation dose resulting in 37% survival) of papillary thyroid cancer cells are within the 1.3–3.2 Gy range [24]. This suggests that the radiation sensitivity of papillary cancer cells is widely distributed.

As shown in Fig. 4, DNA-PKcs in FRO cells moved into the nucleus after irradiation, and DNA-PKcs were still observed in the nuclei of TPC-1 cells. These results indicate that full-length native DNA-PKcs moved to the nucleus after irradiation. Clear foci were observed in the nuclei after γ-ray irradiation, and DNA-PKcs foci were observed in radiation sensitive TPC-1 cells along with a diffused signal. This indicates that cleaved DNA-PKcs remained in the nuclei after irradiation. TPC-1 cells carry small amounts of native DNA-PKcs [9], indicating that native DNA-PKcs aggregated around DSBs for repair and that full-length native DNA-PKcs observed in radiation-resistant FRO cells moved rapidly from the cytoplasm to the nucleus and repaired DSBs. Foci observed in the nuclei of both FRO and TPC-1 cells are considered to be DNA-PKcs bound to DSBs for repair.

Moreover, DSBs were rapidly repaired in FRO cells compared to in TPC-1 cells (Fig. 5). As described in a previous report, DNA-PKcs (NHEJ) functions in the first step of repair. The contribution of fast repair was higher in FRO cells (66%) than in TPC-1 cells (51%). These results indicate that fast repair (NHEJ) contributes to a larger portion of radiation-induced DSB repair in radiation-resistant thyroid cancer cells compared with in radiation-sensitive cells.

In conclusion, the most important aspect of this method is determination of the cellular localization of DNA-PKcs. In radiation-resistant cells, native DNA-PKcs localizes mainly in the cytoplasm, whereas in radiation-sensitive cells, cleaved DNA-PKcs localizes mainly in the nuclei. A limitation of this method is that moderately radiation-sensitive cells cannot be distinguished based on the cellular localization of DNA-PKcs at present.

## CONFLICT OF INTEREST

The authors declare no conflicts of interest associated with this manuscript.

## FUNDING

This work was partly supported by Grants-in-Aid for Scientific Research (C) (No. 23510064) (KAKENHI) Japan. This work was also partly supported by the triangle project of the Network-type Joint Usage/Research Center for Radiation Disaster Medical Science of Hiroshima University, Nagasaki University and Fukushima Medical University.
